# Comment on: Evaluation of three inflammation-associated blood indices for predicting malignancy in thyroid nodules

**DOI:** 10.20945/2359-4292-2026-0069

**Published:** 2026-06-25

**Authors:** Yusuf Öztürk

**Affiliations:** 1 Sakarya University Training and Research Hospital, Department of Endocrinology and Metabolism, Sakarya, Turkey

Dear Editor,

We read with great interest the article by Çatak and cols. entitled “Evaluation of three inflammation-associated blood indices for predicting malignancy in thyroid nodules” ^(1)^. The authors should be commended for their comparative evaluation of systemic inflammatory indices, including the systemic immune-inflammation index (SII), systemic inflammation response index (SIRI), and pan-immune-inflammation value (PIV).

However, we believe that the prognostic assessment presented in the study is methodologically limited. The associations between inflammatory indices and tumor-related features in the malignant cohort were evaluated solely using Pearson or Spearman correlation analyses. While these methods are useful for identifying simple associations, they do not account for potential confounding variables and are insufficient to establish independent prognostic value. In particular, binary clinical outcomes such as lymph node metastasis or advanced stage require regression-based modeling.

Furthermore, the absence of receiver operating characteristic (ROC) analyses and multivariate regression models for prognostic endpoints is noteworthy. Therefore, the lack of significant prognostic findings may reflect methodological limitations rather than a true absence of association.

In contrast, in our study including 376 patients with differentiated thyroid carcinoma, PIV demonstrated significant discriminative ability for distant metastasis and ATA high-risk category, and emerged as an independent risk factor in multivariate analyses ^(2)^. These findings suggest that PIV may reflect tumor aggressiveness within malignant cohorts rather than serving as a diagnostic discriminator.

Additionally, we found that absolute monocyte count (AMC) was significantly associated with TNM stage, ATA high-risk category, and distant metastasis ^(2)^. This observation provides an important perspective on the authors’ suggestion that the monocyte component of PIV may introduce heterogeneity. On the contrary, given the well-established role of monocyte-derived tumor-associated macrophages in tumor progression and metastasis, the monocyte component may represent a key biological driver of the prognostic signal captured by PIV.

In conclusion, we believe that the clinical utility of inflammatory indices depends on the appropriate definition of their intended context. While these markers may have limited value in distinguishing benign from malignant nodules, they may be more useful in identifying aggressive disease and high-risk patients within malignant populations. Accordingly, prognostic evaluations should be performed using appropriate statistical methodologies and clinically relevant endpoints.

## Data Availability

datasets related to this article will be available upon request to the corresponding author.

## References

[1] Çatak M, Koca B, Çetin Z, Başer ÖÖ. (2026). Evaluation of three inflammation-associated blood indices for
predicting malignancy in thyroid nodules. Arch Endocrinol Metab.

[2] Öztürk Y, Kocabaş M, Karaköse M, Kulaksizoğlu M, Karakurt F. (2025). Prognostic significance of the pan-immune-inflammation value
(PIV) in patients with differentiated thyroid carcinoma. Arch Endocrinol Metab.

